# Gestational Diabetes Mellitus Among Pregnant Women Attending Ante-natal Clinic at a Secondary Care Health Facility in Haryana, India

**DOI:** 10.7759/cureus.25452

**Published:** 2022-05-29

**Authors:** Sumit Malhotra, Shashi Kant, Rakesh Kumar, Farhad Ahamed, Suprakash Mandal, Arjun M C, Puneet Misra, Yashdeep Gupta

**Affiliations:** 1 Centre for Community Medicine, All India Institute of Medical Sciences, New Delhi, New Delhi, IND; 2 Centre for Community Medcine, All India Institute of Medical Sciences, New Delhi, New Delhi, IND; 3 Community and Family Medicine, All India Institute of Medical Sciences, Kalyani, Kalyani, IND; 4 Community Medicine and Family Medicine, All India Institute of Medical Sciences, Bhubaneswar, Bhubaneswar, IND; 5 Department of Endocrinology and Metabolism, All India Institute of Medical Sciences, New Delhi, New Delhi, IND

**Keywords:** north india, secondary level care hospital, gdm, ballabgarh, gestational diabetes

## Abstract

Introduction: Gestational diabetes has serious health effects during pregnancy and childbirth. We estimated the occurrence of gestational diabetes mellitus (GDM) among pregnant women in a secondary care hospital in Haryana.

Methods: It was a hospital-based cross-sectional study, done in an ante-natal clinic (ANC) at a sub-district hospital (SDH), Faridabad district of Haryana, India. Eligible pregnant women attending the ANC clinic were recruited. An oral glucose tolerance test (OGTT) with 75 g of glucose was done with a collection of blood for fasting blood sugar (FBS) and two-hour post-OGTT blood glucose. A pre-tested semi-structured interview schedule was administered. Both the modified International Association of the Diabetes and Pregnancy Study Groups criteria (IADPSGC) and the Diabetes in Pregnancy Study Group of India (DIPSI) criteria were used. Data were presented as percentages, means, standard deviation, and 95% confidence interval (CI). Bi-variable and multi-variable logistic regressions were done. The level of significance was set at 0.05.

Results: Of the 623 eligible participants, 66.1% were within the 20-25 age group. The GDM was found in 14.1% (95%CI: 11.5-17.1) participants as per modified IADPSG criteria and 6.7% (95%CI: 4.9-9.0) participants as per DIPSI criteria, respectively. Increasing age [adjusted odds ratio (AOR): 1.24 (95% CI: 1.05-1.47), p=0.008] and increasing years of schooling [AOR: 1.19 (1.01-1.41), p=0.032] were significantly associated with GDM by DIPSI criteria. Family history of DM was also found to have an increased odds with GDM using modified IADPSG criteria [AOR 2.87 (95% CI: 1.09-7.54), p=0.032].

Conclusion: Considerable proportion of pregnant women were found to have GDM in a Sub-district hospital at Ballabgarh in north India. The study highlighted the need and generated evidence about the feasibility of GDM screening utilizing routine staff in a secondary care facility.

## Introduction

Gestational diabetes mellitus (GDM) is defined as glucose intolerance with onset or first recognition during pregnancy, which is not diagnostic of overt diabetes [1]. Although asymptomatic in its clinical course, GDM, if not timely identified and adequately managed, may lead to serious adverse obstetric and neonatal outcomes [2,3]. In the long run, both mother and child have higher chances of developing type 2 diabetes mellitus, and babies are more likely to become obese later in life [4,5].

The global prevalence of GDM varies widely, depending on population characteristics (e.g., maternal age, socioeconomic status (SES), race/ethnicity, or body composition), screening methods, and diagnostic criteria. As per the International Diabetes Federation, an estimated 21.1 million (16%) live births had some form of hyperglycemia in pregnancy in the year 2019, with the highest prevalence (28%) estimated in South East Asia (SEA) [6]. As per a systematic review, the prevalence of GDM was found to be 10.1% in SEA [7].

The International Association of Diabetes and Pregnancy Groups (IADPSG) proposed a set of criteria to diagnose GDM. According to these criteria, the diagnosis of GDM is made if at least one value of plasma glucose concentration is equal to or exceeds the thresholds of 92, 180, and 153 mg/dl for fasting, one-hour, and two-hour post glucose load glucose values, respectively, after performing a 75-g oral glucose tolerance test (OGTT) [8]. The World Health Organization accepted these criteria for screening for GDM [9]. The Diabetes in Pregnancy Study Group of India (DIPSI) criteria were used in guidelines on diagnosis and management of gestational diabetes mellitus by the Government of India and it recommended OGTT at 24-28 completed weeks of gestation to diagnose GDM [10]. As per DIPSI criteria, two-hour OGTT ≥ 140 mg/dl is considered GDM [11].

Increasing mechanization and urbanization of rural populations have been associated with an increased occurrence of type 2 diabetes in rural areas of India [12]. Undetected and untreated GDM will add to the increasing burden of non-communicable diseases in the country [13]. However, adequate information is not available about the study area to take necessary action to prevent and minimize short-term and long-term maternal and fetal morbidity associated with GDM. The current study was done to ascertain the occurrence of GDM and study the socio-demographic and other factors associated with it among pregnant women with 24-28 completed weeks of gestation (POG) attending the ante-natal clinic (ANC) in a secondary healthcare facility in Haryana, India by modified IADPSG and DIPSI criteria. We additionally compared the results obtained through the two criteria.

## Materials and methods

Study design

This is a hospital-based cross-sectional study.

Study participants

Pregnant women with 24-28 completed weeks of gestation attending an antenatal clinic (ANC) at a a sub-district hospital (SDH) in Ballabgarh, Faridabad, Haryana were recruited for the study. Pregnant women who were suffering from chronic renal, pancreatic, and other severe illnesses; receiving steroids, nicotinic acid, or other medication likely to cause dysglycemia; and those with previously diagnosed diabetes mellitus were excluded from the study.

Study setting

The study was conducted in an ANC at Ballabgarh SDH. This facility is a 50-bed secondary care hospital, which was part of the Comprehensive Rural Health Services Project under the Centre for Community Medicine, All India Institute of Medical Sciences (AIIMS), New Delhi. The hospital provides services to Ballabgarh town in the district of Faridabad, with a population of around 190,000, and nearby areas of the district of Palwal, Haryana. It also caters to the health needs of nearly 100,000 people residing in 28 villages of the Ballabgarh block of the district of Faridabad. The daily new outpatient attendance is around 500 clients. The ANC clinic functions on Wednesday, Thursday, and Friday afternoons with an average daily attendance of at least 50 pregnant women. The study was conducted during the period from January to March 2019.

Methodology

Eligible pregnant women attending the ANC clinic at SDH Ballabgarh were given an appointment for an OGTT test and asked to come after overnight fasting. A nurse posted at the ANC clinic explained the whole procedure in detail to all eligible study participants and obtained consent for participation. On the scheduled dates when the study participants came for OGTT, a pre-tested semi-structured interview schedule was administered to all eligible pregnant women to collect information on their socio-demographic profile, relevant medical and obstetric history, previous history of GDM, family history of diabetes mellitus, and awareness regarding GDM. A modified BG Prasad socio-economic scale was used to assess the socio-economic status of study participants [14]. Anthropometric measurements including body weight (to the nearest 0.1 kg; Equinox Personal Weighing Scale-Digital EQ-EB-9300) and height (to the nearest 0.1 cm; Seca 213 portable Stadiometer) were recorded. Blood pressure was recorded thrice at an interval of five minutes in a seating position in the non-dominant arm (to the nearest 02 mmHg) using a standard digital sphygmomanometer (Omron HEM 8711). The mean of the three readings was taken as the blood pressure. These measurements were done by a single trained nurse, and standardization, along with quality assurance measures, was undertaken through supportive supervision by the study team. 

Sample size

A minimum of 616 pregnant women was required in the study assuming a prevalence of GDM of 13.9% [15], a two-tailed alpha error of 5%, power of 90%, and 20% of relative precision. All pregnant women of 24-28 weeks of gestation, presenting with antenatal OPD of SDH Ballabgarh during the study period, were offered to be part of the study.

Oral glucose tolerance test

All eligible study participants were subjected to an OGTT with 75-g anhydrous glucose powder. The glucose powder was dissolved in 250-300 ml of water and pregnant women were asked to consume it in five minutes. Two millilitres (ml) of blood samples were taken aseptically from the ante-cubital vein twice for estimation of fasting and two-hour post-glucose blood sugar levels in a biochemical analyzer (Biolis 24i Premium) and HbA1C in another automated analyzer (Adams Arkray, Model HA-8180, Japan that estimates using high-performance liquid chromatography). If the pregnant woman vomited within 30 minutes of consumption of glucose, the OGTT test was repeated on the next ANC day of the clinic or the following week. This was allowed a maximum of three attempts. In the case of vomiting after 30 minutes, the OGTT test was completed on the same day, as per the guidelines of the government of India [10]. Instruments were calibrated regularly for the purpose of quality assurance.

Operational definitions

Modified IADPSG Criteria

Any one value of plasma glucose concentration equal to or exceeding the thresholds of 92 and 153 mg/dl (for fasting and two-hour post glucose load values, respectively), after performing a 75 g OGTT, was considered as GDM.

DIPSI Criteria

A blood glucose level of ≥140 mg/dl two hours after the consumption of 75 mg of anhydrous glucose was considered GDM.

Ethics

Ethical approval was obtained from the Institutional Ethics Committee, AIIMS, New Delhi (vide Letter Reference No. IEC/649/11/2018). Informed written consent was taken from each study participant after reading and explaining the participant information sheet to them in their local language. Pregnant women diagnosed with GDM were managed as per national guidelines of the government of India by the obstetrician in our study hospital [10].

Statistical analysis

The data were entered in a Microsoft Excel sheet (Microsoft® Corp., Redmond, WA) and analyzed with the help of STATA 12 statistical software (StataCorp LLC, TX, USA). Categorical data were presented as percentages (%). Means and standard deviations were used to present normally distributed data. Binary logistic regression analysis (stepwise method) was used to evaluate the independent associations of various factors with the prevalence of GDM. The multivariable logistic analysis was done for all the variables after the bi-variable analysis. A "p" value of less than 0.05 was considered statistically significant.

## Results

A total of 623 out of 690 pregnant women were recruited for the study and invited to participate during the study duration, with a response rate of 90%. The mean (SD) age of the study participants was 23.8 (3.5) years (95%CI: 23.5-24.1). Two-thirds of the study participants were in the age group of 20-25 years (66.1%). The mean (SD) number of years of schooling among study participants was 10.0 (4.6) years (95%CI: 9.6-10.4). Almost all (95%) of the study participants were housewives. The median (interquartile range) per capita family income was INR 3400 (2500-5000). Details of the socio-demographic characteristics of study participants have been described in Table 1.

**Table 1:** Distribution of study participants by selected variables and GDM status using modified IADPSG criteria and DIPSI criteria GDM: gestational diabetes mellitus, IADPSG: International Association of the Diabetes and Pregnancy Study Groups, DIPSI: diabetes in pregnancy study group of India, LSCS: lower (uterine) segment caesarean section. The percentages depicted are row-wise percentages.

S.no	Variables	Groups	Total number in each category (n)	Modified IADPSG^1^ GDM (n=88)	DIPSI^2^ GDM (n=42)
				Number (%)	Number (%)
1.	Age in years	<20	57	5 (8.77)	3 (5.3)
		20–25	412	61 (14.81)	24 (5.8)
		26–30	128	18 (14.06)	11 (8.6)
		>30	26	4 (15.4)	4 (15.4)
2.	Years of schooling	≤10	346	49 (14.16)	19 (5.5)
		>10	277	39 (14.08)	23 (8.3)
3.	Occupation	Housewife	590	84 (14.24)	40 (6.7)
		Others	33	4 (12.12)	2 (6.1)
4.	Per capita family income (as per BG Prasad’s socioeconomic scale) amount in Indian rupees	>7008	71	10 (14.1)	0 (0.0)
		3504–7007	237	35 (14.7)	3 (7.1)
		2102–3503	196	27 (13.8)	10 (23.8)
		1051–2101	106	12 (11.3)	21 (50.0)
		≤1050	13	4 (30.7)	8 (19.1)
5.	Gravida	1	200	31 (13.48)	16 (7.0)
		2–3	321	45 (14.02)	23 (7.2)
		≥4	72	12 (16.67)	3 (4.2)
6.	Past H/O stillbirth (n=393)	Yes	9	4 (44.4)	0 (0.0)
		No	384	53 (13.8)	9 (2.4)
7.	H/O LSCS^3^ (n=393)	Yes	44	6 (13.6)	4 (9.1)
		No	349	51 (14.6)	22 (6.3)
8.	Family H/O diabetes in first-degree relatives	Yes	62	12 (19.4)	5 (8.1)
		No	561	76 (13.6)	37 (6.6)
9.	Presence of any high-risk factor in present pregnancy	Yes	280	42 (15)	13 (4.6)
		No	343	46 (13.4)	29 (8.5)
11.	HbA1c level	≥5.7%	14	5 (35.7)	2 (4.4)
		<5.7%	609	83 (13.6)	40 (6.9)

Almost one-third of the study participants were primigravida (36.9%). The mean (SD) period of gestation of study participants was 25.6 (1.7) weeks. Among multigravida study participants (n=393), nine study participants (2.3%) reported stillbirths in the last pregnancy. Around one in every ten study participants (11.2%) had undergone cesarean delivery in their last pregnancy. Almost 10% of the study participants reported a history of diabetes mellitus among first-degree family relatives. A total of 280 (44.9%) study participants reported at least one high-risk factor (as mentioned in the study appendix, like age <18 or >35 years, weight <45 kg, height <145 cm, grand multipara, pre-eclampsia, severe anaemia, antepartum haemorrhage, multiple pregnancies, abnormal presentation, etc.) in the current pregnancy, whereas 26.0% of pregnant women reported having at least one high-risk factor in the last pregnancy. Only two pregnant women reported that they were diagnosed with a case of GDM in an earlier pregnancy. A total of 295 pregnant women recalled the birth weight of the youngest babies. The mean (SD) weight of the youngest babies was 2772.0 g (593.5; n=295). A history of big babies (birth weight ≥ 3500 g) was reported by 53 (8.5%) study participants (n=295). The mean height (SD) and weight (SD) of study participants were 150.8 (6.3) cm and 54.9 (9.3) kg, respectively. Pre-pregnancy weights were available for 502 study participants. So, we calculated pregnancy weight gain (current weight - pre-pregnancy weight) for these participants. The median (IQR) pregnancy weight gain by study participants was 5.0 (3.0-7.1) kg.

Out of a total of 623 participants, 77 (12.4%) had fasting plasma glucose more than equal to 92 mg/dl with a mean value of 96.6 mg/dl (SD= 5.35, 95% CI: 95.4 -97.9). After two hours of the oral glucose challenge, 17 (2.7%) participants had plasma glucose levels more than equal to 153 mg/dl with a mean value of 168.1 mg/dl (SD=12.3, 95% CI: 161.8-174.4). Only six (0.9%) participants had both fasting plasma glucose and two-hour post-OGTT plasma glucose more than equal to 92 mg/dl and 153 mg/dl, respectively. As per the modified IADPSG criteria, the prevalence of GDM among study participants was 14.1% (95%CI: 11.5-17.1). As per the DIPSI guideline, the prevalence of GDM among study participants was 6.7% (95%CI: 4.9-9.0). For all other analyses, both the modified IADPSG and the DIPSI classification for GDM have been followed in this study. The number of positive cases that came by the modified IADPSG and DIPSI criteria was significantly different with a value of p<0.001 (Table 2) and the value of kappa was 0.31 (p<0.001).

**Table 2:** Comparison of the positive and negative participants for gestational diabetes mellitus as per modified IADPSG and DIPSI criteria Pearson chi-square (1) = 68.7059, p < 0.001.

DIPSI criteria	Modified IADPSG criteria		Total
	Positive	Negative	
Positive	24 (57.14)	18 (42.86)	42
Negative	64 (11.02)	517 (88.98)	581
	88	535	623

In the multivariable analysis (Table 3), as per the modified IADPSG criteria, family history of DM in first-degree relatives had three times higher odds of having GDM [Adjusted Odds Ratio 2.87 (95% CI: 1.09-7.54), p=0.032].

**Table 3:** Factors associated with GDM among study participants using bi-variable and multivariable logistic regression as per modified IADPSG criteria LSCS: lower (uterine) segment caesarean section, GDM: gestational diabetes mellitus, IADPSG: Modified International Association of the Diabetes and Pregnancy Study Group Criteria, CI: confidence intervals, Reference - comparison category.

Sno	Variables	Groups	Crude odds ratio (95%CI)	p-value	Adjusted odds ratio (95%CI)	p-value
1.	Age		1.03 (0.96–1.09)	0.419	1.01 (0.91–1.13)	0.743
2.	Years of schooling		0.98 (0.93–1.03)	0.376	0.98 (0.91–1.07)	0.771
3.	Occupation	Housewife	Reference	0.734	Reference	0.704
		Others	0.83 (0.28–2.42)		1.30 (0.32- 5.18)	
4.	Per capita family income		0.99 (0.98–1.00)	0.855	0.99 (0.98–1.00)	0.969
5.	Gravida	Primigravida	Reference	0.679	0.99 (0.68–1.45)	0.98
		Multigravida	1.08 (0.68–1.74)			
6.	History of LSCS (n=393)	No	Reference	0.862	Reference	0.813
		Yes	0.92 (0.37–2.30)		1.13 (0.40–3.20)	
7.	Family history of DM in first-degree relatives	No	Reference	0.216	Reference	0.032
		Yes	1.53 (0.78–3.0)		2.87 (1.09–7.54)	
9	Birth weight of last born baby		1.0 (0.99–1.01)	0.09	1.00 (0.99–1.01)	0.061

However, as per the DIPSI criteria (Table 4), two variables were found to be significantly associated with GDM. On multivariable analysis, increasing age was found to have 1.24 times higher odds [adjusted OR: 1.24 (95%CI: 1.05-1.47) of developing GDM (p=0.008). Also, increasing years of schooling were found to have 1.19 times higher odds [adjusted OR 1.19 (95% CI: 1.01-1.41, p=0.032)] of developing GDM. The association between GDM and other factors was found to be statistically non-significant.

**Table 4:** Factors associated with GDM among study participants using bi-variable and multivariable logistic regression as per DIPSI criteria LSCS: lower (Uterine) segment caesarean section, GDM: gestational diabetes mellitus, DIPSI: Diabetes in Pregnancy Study Group of India Criteria, CI: confidence intervals, Reference - comparison category.

Sno	Variables	Groups	Crude odds ratio (95%CI)	p-value	Adjusted odds ratio (95%CI)	p-value
1.	Age		1.1 (1.03–1.21)	0.005	1.24 (1.05–1.47)	0.008
2.	Years of schooling		1.08 (1.00–1.16)	0.048	1.19 (1.01–1.41)	0.032
3.	Occupation	Housewife	Reference	0.873	Reference	0.898
		Others	0.89 (0.20–3.8)		1.17 (0.19–7.37)	
4.	Per capita family income		0.99 (0.98–1.00)	0.968	0.99 (0.98–1.00)	0.311
5.	Gravida	Primigravida	Reference	0.140	Reference	0.912
		Multigravida	0.62 (0.33–1.16)		0.96 (0.47–1.97)	
6.	History of LSCS (n=393)	No	Reference	0.748	Reference	0.785
		Yes	0.78 (0.17–3.47)		0.74 (0.08-6.6)	
7.	Family history of DM in first-degree relatives	No	Reference	0.003	Reference	0.139
		Yes	3.18 (1.47–6.83)		2.9 (0.71-12.11)	
9	Birth weight of last born baby		0.99 (0.98–1.0)	0.430	0.99 (0.98 – 1.0)	0.434

## Discussion

A total of 623 pregnant women were screened to determine the prevalence of GDM and its associated factors. The prevalence of GDM was 14.1% by modified IADPSG criteria and 6.7% by DIPSI criteria. A wide range of prevalence of GDM (1-42%) has been reported by studies conducted in different parts of India [16]. Major reasons for such a wide variation in reported prevalence were the different diagnostic criteria used in the studies and differences in the socio-demographic profile of the study population. The American Diabetic Association (ADA), the IADPSG, and the DIPSI are various professional bodies that have suggested different cut-off values for diagnosing GDM in pregnant women. A study conducted in Gujarat reported that the estimated prevalence of GDM using capillary blood was higher in comparison to the estimated prevalence of GDM using venous blood (20.4% vs 11.5%) [17]. In our study, we used a venous blood sample for the estimation of blood glucose. This might be a reason for the low prevalence of GDM in our study. Our prevalence estimate of 14% using IADPSG criteria matches with the prevalence estimate reported in another hospital-based study from a South Indian setting utilizing the same criteria [18].

Both the IADPSG and DIPSI criteria have been used in this study. Internationally, IADPSG criteria are more widely accepted, though the government of India has accepted DIPSI guidelines for use in programmatic settings due to feasibility and logistical concerns. In our study, a higher prevalence of GDM was obtained using IADPSG criteria. A meta-analysis of Indian studies that pooled estimates for GDM using IADPSG criteria reported a relatively higher prevalence than other criteria at 19.2% (95% CI: 15.5, 23.6) [16]. The same study reported the pooled estimate emanating from studies that utilized DIPSI criteria as 7.4% (95% CI: 5.2, 10.2). Another systematic review that included studies determining the prevalence of GDM in South Asian countries also reported a higher prevalence estimate of GDM using IADPSG criteria (20.9%, 95% CI: 17.3, 24.6) compared to studies that used DIPSI criteria (8.3%, 95% CI: 5.7, 10.9) [19]. In our study also, we found that the prevalence of GDM was higher with IADPSG criteria than with DIPSI. The level of agreement between the two criteria in the study was found to be poor (kappa = 0.31). A study of 144 South Indian women reported sensitivity and specificity as 45% and 87%, respectively [20].

We found that pregnant women of increasing age had a higher chance of having GDM. Higher maternal age is a known factor associated with GDM. Studies conducted in different parts of India also reported similar findings [16,21]. With increasing age, individuals may become less physically active and develop other risk factors as well. This might be the possible reason for such an association.

There was a statistically significant association between a positive family history of diabetes among first-degree relatives and increased odds of GDM on bi-variable and multivariable analysis. A family history of diabetes has some genetic predisposition to the development of GDM [22,23]. Our study result is consistent with the previous study done by Rajput et al. in a tertiary care hospital in Haryana among 607 ANC mothers from 24 to 28 weeks of gestation where the crude OR was 2.35 (95% CI: 0.99-5.6, p<0.05) though it was not significant in multivariable analysis [24]. A meta-analysis was done by Lee et al. among the Asian population, which also showed an OR of 2.77 (95% CI: 2.22-3.47), which is similar to our study findings [19].

Some Indian studies reported an increased chance of GDM among study participants with higher socioeconomic status [24], mostly due to a more sedentary lifestyle and a higher pre-pregnancy weight among study participants from higher SES. Our study did not find this association significant. One study done in China did not find any association between SES and GDM [25]. In fact, one study from China reported an inverse association between SES and GDM [26]. In many countries, people from higher socioeconomic strata are found to be more physically active and lead a healthy lifestyle [27]. This might be the possible explanation for an inverse association between SES and GDM in their studies.

Years of schooling were found to be associated with an increased odds of GDM. Though the longer duration of education may also be associated with a higher probability of higher maternal age, a modern lifestyle, and less physical activity among study participants, our study did not find the association. A study conducted in Rotterdam, the Netherlands, discovered that a lower educational level was associated with a higher prevalence of GDM. However, the study also reported that a large part of the observed increased risk of GDM in the lower educated group was due to relatively higher rates of overweight and obesity among those women [28].

This was a hospital-based study, yet in a secondary care facility that was closer to community settings. Blood was collected by a trained nurse; this added to the strength of our study. Venous blood was collected for blood glucose estimation. All quality control measures were followed for laboratory investigations, which minimized the measurement errors. Our study had a few limitations also. This was a single-centre study in a peri-urban setting in the northern part of the country. The findings might not be generalizable to other settings, and thus a multi-centric study would be needed to establish prevalence in different regions of the country. We did not measure blood glucose during the first trimester of pregnancy and kept a single visit during the last 24-28 weeks as the basis for estimating prevalence. Also, our study was not powered sufficiently to ascertain associations with all factors, as primarily our study was designed to ascertain the occurrence of GDM among pregnant women attending a secondary care hospital. We also did not include anthropometry and blood pressure measurements for determining associations with GDM and future studies in this regard can study these factors in-depth and measure these associations in a larger sample of pregnant women.

## Conclusions

We have reported the levels of GDM using two criteria-one that is more internationally accepted and the other that is recommended by the Government of India for incorporation in the programmatic settings. It has been recommended now that an OGTT be performed for all pregnant women visiting ANC clinics in routine district health systems within India. However, OGTT tests are not being done routinely, even in secondary care hospitals. With such a high burden of GDM among pregnant women, all health care settings need to include OGTT in essential obstetric care packages. Our study has demonstrated that it is feasible to perform this in a government secondary care facility. In our sample, 14% and 7% of pregnant women attending the ANC clinic at SDH Ballabgarh had GDM as per the IADPSG and DIPSI criteria, respectively. The latter criteria under-reports the prevalence of GDM. Increasing age, higher years of education, and a positive family history of diabetes were associated with an increased risk of GDM. Future studies should also track prospectively the maternal and neonatal outcomes in these women detected with GDM.

## Appendices

         Comprehensive Rural Health Services Project Ballabgarh

                     Centre for Community Medicine, All India Institute of Medical Sciences, New Delhi

**Table 5:** Study questionnaire

S.no.	Question	Responses
1	Name	
2	Age (in completed years)	
3	Years of schooling	
4	Occupation	1. Housewife
		2. Government Job
		3. Private Job
		4. Self-employed
		5. Others (Specify)
5	Obstetric history	G- P- L- A- S-
6	Last menstrual period (LMP)	
7	Socio-economic class (BG Prasad Scale) (monthly per capita income). Write monthly family income (in Rs.). The total number of family members:	I ≥ Rs. 6276
		II = Rs. 3139-6276
		III= Rs. 1883-3138
		IV= Rs. 942-1882
		V ≤ Rs.942
8	High-risk factors in present pregnancy	0. None
		1. Age <18 or >35 years
		2. Weight <45 kg
		3. Height <145 cm
		4. Grand multigravida (≥4)
		5. Pre-eclampsia (BP>140/90 mm hg, edema feet)
		6. Severe anaemia (Hb<7 gm%)
		7. Bleeding PV (APH)
		8. Multiple pregnancies (twins)
		9. Abnormal presentation
		10. Rh incompatibility
		11. Others (Specify)
9	High-risk factors in a past pregnancy	0. None
		1. Age <18 or >35 years
		2. Systemic disease (cardiac ds, renal ds, HTN, DM, TB)
		3. Previous LSCS
		4. Neonatal death/stillbirth
		5. Previous difficult labour
		6. Rh incompatibility
		6. Bad obstetric history
		7. Others (Specify)
10	History of diabetes mellitus in first-degree relatives (parents and siblings)	0=No
		1=Yes
11	Were you diagnosed with gestational diabetes mellitus (GDM) in your previous pregnancy? (Skip, in PRIMI Gravida)	0=No
		1=Yes
12	Did you ever undergo LSCS in your last pregnancies? (Skip, in PRIMI Gravida)	0=No
		1=Yes
13	What was the birth weight of your last delivered child? (Skip, in PRIMI Gravida)	
14	Pre-pregnancy weight (first-trimester weight) in kg	1. Available-Wt: kg
		2. Not available
15	Anthropometry (at the time of interview)	Weight (in kg):
		Height (in cm):
16	Blood pressure (mm/Hg)	
17	Blood sugar (venous) (in mg/dl)	Fasting:
		2-hr after Glucose:
18	HbA1C	
